# Expansion and application of dye tracers for measuring solid food intake and food preference in *Drosophila*

**DOI:** 10.1038/s41598-021-99483-7

**Published:** 2021-10-08

**Authors:** Brandon C. Shell, Yuan Luo, Scott Pletcher, Mike Grotewiel

**Affiliations:** 1 Department of Human and Molecular Genetics, School of Medicine, Virginia Commonwealth University, Richmond, VA 23298 USA; 2 Department of Molecular and Integrative Physiology and Geriatrics Center, University of Michigan, Ann Arbor, MI 48109 USA

**Keywords:** Biological techniques, Physiology

## Abstract

The *Drosophila* model is used to investigate the effects of diet on physiology as well as the effects of genetic pathways, neural systems and environment on feeding behavior. We previously showed that Blue 1 works well as a dye tracer to track consumption of agar-based media in *Drosophila* in a method called Con-Ex. Here, we describe Orange 4 as a novel dye for use in Con-Ex studies that expands the utility of this method. Con-Ex experiments using Orange 4 detect the predicted effects of starvation, mating status, strain, and sex on feeding behavior in flies. Orange 4 is consumed and excreted into vials linearly with time in Con-Ex experiments, the number of replicates required to detect differences between groups when using Orange 4 is comparable to that for Blue 1, and excretion of the dye reflects the volume of consumed dye. In food preference studies using Orange 4 and Blue 1 as a dye pair, flies decreased their intake of food laced with the aversive tastants caffeine and NaCl as determined using Con-Ex or a more recently described modification called EX-Q. Our results indicate that Orange 4 is suitable for Con-Ex experiments, has comparable utility to Blue 1 in Con-Ex studies, and can be paired with Blue 1 to assess food preference via both Con-Ex and EX-Q.

## Introduction

Diet and dietary intake have long been associated with several chronic disease states including obesity, diabetes, heart disease, stroke and cancer. The *Drosophila* (fruit fly) model has become a leading experimental platform for investigating genetic and environmental influences on feeding behavior as well as the impact of diet on many physiological and pathophysiological responses. For example, the fly model has been used to examine protein valuation^1^, compensatory feeding^2^, ethanol consumption^3^, effects of starvation^4^, the relationship between food intake and sleep^5,6^, effect of diet on mating^7^, the relationship between diet and insulin resistance^8^, nervous system control of behavior in response to hunger^9^, the effect of diet on subsequent food intake^10^, and the effect of diet on ethanol sedation^11^. Diet also affects rodent behaviors such as ethanol consumption^12^ as well as anxiety-like behavior and visual-spatial memory^13^. The fly model has the potential to greatly expand our understanding of the relationships between diet and disease, as well as the mechanisms that drive food consumption and food choice.

Given its popularity for studies on diet, several methods have been developed to assess consumption of agar-based food media in flies. We developed Con-Ex as a method using Blue 1 as a dye tracer for solid food medium in which excreted dye reflects the volume of food medium flies have consumed^14^. A related method, called EX-Q, also uses Blue 1 as a dye tracer for solid food consumption like Con-Ex, but provides dye-labeled media to flies in smaller containers^15^. The smaller containers of media used in EX-Q reduce the amount of dye excreted on (i.e., lost to) the surface of the food media, thereby increasing the fraction of consumed dye measured as excreted dye^15^. Radioactive tracers such as [α-32P]dCTP have also been used to label solid food media in flies, with consumption estimated by internal accumulation of the tracer in fly tissues^16^. A major advantage of the radioactive tracer methods is their sensitivity, whereas major advantages of the dye-based Con-Ex and EX-Q methods is their simplicity, low cost and ease of use.

A key aspect of dietary intake is food preference or food choice. Food preference has major implications for human health and wellness including a role in obesity in adults and children, psychological health and even financial status^17^. Understanding mechanisms that drive food choice could therefore ultimately lead to mitigation strategies for a wide range of disease-like states. In addition to its utility for investigating consumption of a single food type, the *Drosophila* model should be well suited for investigating food preference when presented with multiple food options. Accordingly, methods exist for assessing food preference when flies are presented with solid, agar-based media labeled with different dyes (e.g., references^18,19^), solid media labeled with a single radioactive tracer^20^, solid media tracked by oligonucleotide ingestion^21^, or liquid media via CAFE^3^. These methods have demonstrated utility and their advantages include sensitivity^3,18–21^ as well as ease of measuring the tracers themselves^3,18–20^, but their implementation might be limited in some experimental settings due to the use of non-standard housing conditions or media^3,18,19^, inability to quantitate the volume of media consumed^18,19^, potential challenges with secure handling of radioactive flies^20^, difficulty in explicitly measuring consumption of different media simultaneously^18–20^, or use of sophisticated detection methods^21^. To the best of our knowledge, no dye-based methods have been described for assessing food preference in flies when they are provided with solid agar-based media under largely standard housing conditions.

Here we report our studies on Orange 4 as an additional dye for use in Con-Ex experiments. Orange 4 can be used as an alternative to Blue 1 in Con-Ex or to confirm findings obtained with Blue 1 in Con-Ex. Additionally, Orange 4 can be paired with Blue 1 to monitor food preference in both the Con-Ex and EX-Q methods. Our findings establish Orange 4 as a dye suitable for Con-Ex and demonstrate the utility of Orange 4 and Blue 1 as a dye pair in the analysis of food preference in flies consuming solid media.

## Results

We previously described Blue 1 as a dye suitable for use in Consumption-Excretion (Con-Ex) studies for monitoring intake of agar-based food media in *Drosophila*^14^. In Con-Ex studies, flies are provided with an agar-based food medium labeled with dye for prescribed amounts of time, the food medium with dye is consumed, excretion products containing dye accumulate inside the vials housing flies, and the amount of dye excreted is quantified by spectrophotometry^14^. Although it is possible to measure the amount of internal dye inside flies (INT) and the amount of dye deposited on the surface of the food medium (ExMedium) in Con-Ex studies, this approach is time-intensive and measuring ExVial (the amount excreted into the vial) alone leads to comparable conclusions compared to measuring ExVial plus INT plus ExMedium^14^. Additionally, more dye is recovered as ExVial than as INT or ExMedium^14^. We therefore used ExVial as a measure of Con-Ex throughout the studies described here unless otherwise noted.

We reasoned that identifying additional dyes for use in Con-Ex studies could provide several experimental advantages such as the ability to concurrently monitor consumption of different media in food preference studies. We therefore searched for additional dyes available from commercial vendors that were water soluble, non-hazardous, non-irritating upon skin contact, and had individual peak absorbances at wavelengths across the visible spectrum. We selected 29 dyes as candidates for use in Con-Ex studies (Table S1, provided as supplementary information).

Two important properties of dyes suitable for use in Con-Ex studies are (i) they are readily detectable in ExVial samples and (ii) they do not substantially inhibit or increase consumption-excretion of food media at concentrations that allow their detection. We determined whether the 29 candidate dyes in Table S1 had these properties by measuring ExVial after providing mated control GL females with our standard food medium (2Y10S3C, 2% yeast, 10% sugar, 3% cornmeal in 1% agar, see “Materials and methods” for details) labeled with 0.5, 1.0 or 2.0% (w/v) of each dye for 24 h. As expected, the amount of each dye observed in ExVial samples varied depending on the dye used (Supplementary Fig. S1). We detected strong absorbance of Orange 4 (Supplementary Fig. S1a), Yellow 10 (Supplementary Fig. S1a), Patent Blue (Supplementary Fig. S1b), Yellow 6 (Supplementary Fig. S1b), Acid Blue 3 (Supplementary Fig. S1c) and Light Green SF (Supplementary Fig. S1d) in ExVial samples (red arrows) as a function of the dye concentration used. We continued characterizing these six lead candidate dyes as described below.

The dyes Cochineal Red A (Supplementary Fig. S1b), Orange G (Supplementary Fig. S1d), Bromophenol Red (Supplementary Fig. S1e) and Red 6 (Supplementary Fig. S1f) had moderate absorbance values in ExVial samples and absorbance of these dyes increased with dye concentration. Although these four additional dyes are potentially useful in Con-Ex studies, they were not characterized further in this study. All other dyes tested had low absorbance values in ExVial samples (Supplementary Fig. S1) and are not considered hereafter.

**Figure 1:**
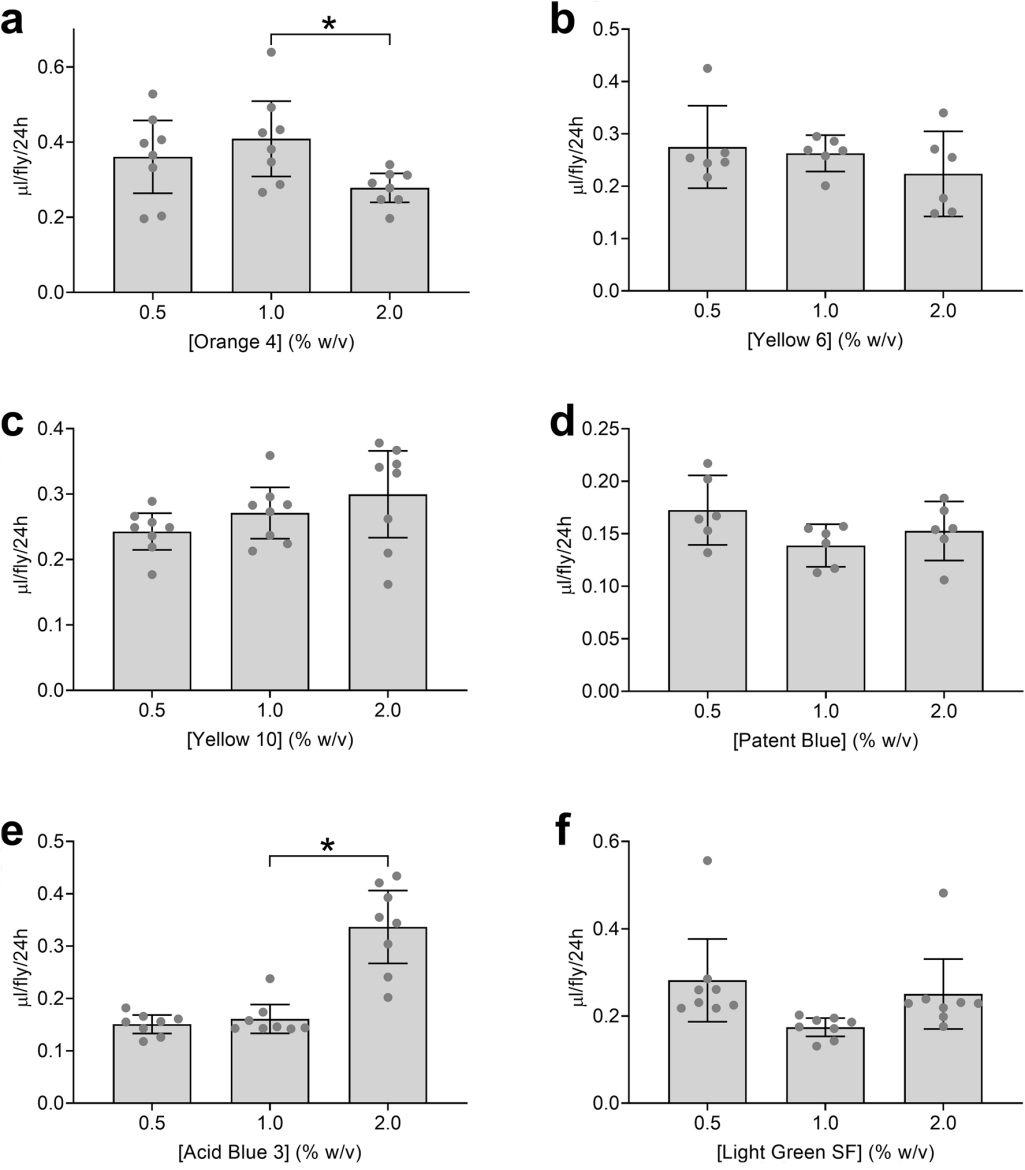
Six lead candidate dyes in Con-Ex: dye concentration in standard medium. Data are ExVial values from mated GL females provided with standard media (2Y10S3C) for 24 h containing the indicated concentrations of (**a**) Orange 4, (**b**) Yellow 6, (**c**) Yellow 10, (**d**) Patent Blue, (**e**) Acid Blue 3 and (**f**) Light Green SF (n = 6–8). Data are ExVial volumes determined from absorbance values in Supplementary Fig. S1. The concentration of Orange 4 (panel A) and Acid Blue 3 (panel E) had an overall effect on ExVial (individual one-way ANOVAs; Orange 4, p = 0.0489; Acid Blue 3, p < 0.0001). Concentrations of the other dyes tested did not have overall effects on ExVial (individual one-way ANOVAs; Yellow 6, p = 0.3852; Yellow 10, p = 0.1156; Patent Blue, p = 0.1183; Light Green SF, p = 0.0594). ExVial values in flies provided with 2% Orange 4 and 2% Acid Blue 3 were significantly different than in flies provided with 1% of the same dyes (*Bonferroni’s; Orange 4, p = 0.0324; Acid Blue 3, p < 0.0001), but not in flies provided with 0.5% of the dyes (Bonferroni’s; Orange 4, p = 0.6946; Acid Blue 3, p > 0.9999).

We determined ExVial volumes from the absorbance data in Supplementary Fig. S1 for each of the six lead candidate dyes as described previously^14^. In these initial experiments, we found that the concentration of Orange 4 (Fig. 1a) and Acid Blue 3 (Fig. 1e) significantly affected ExVial volumes at the highest concentration used. In an expanded repeat experiment, the concentration of Orange 4 did not affect ExVial (Supplementary Fig. S2), suggesting that this dye at a concentration up to 2% does not have a reproducible effect on Con-Ex. We found no significant effect of concentration of the other four lead candidate dyes on ExVial (Fig. 1b, Yellow 6; Fig. 1c., Yellow 10; Fig. 1d, Patent Blue; Light Green SF, Fig. 1f). All subsequent studies were performed with dyes at 1% to ensure they were used below concentrations that affect ExVial.

**Figure 2:**
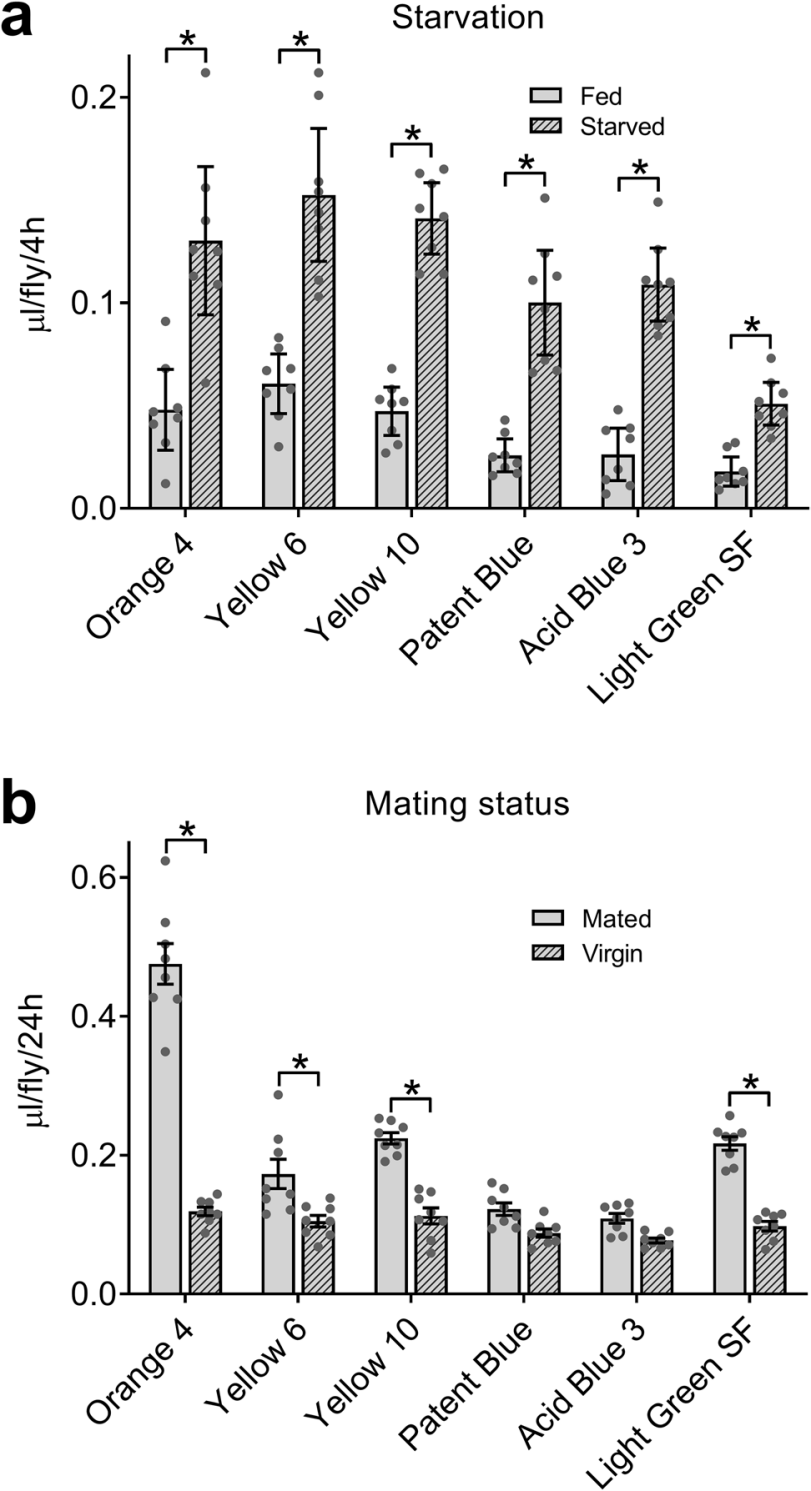
Six lead dyes in Con-Ex: response to starvation and mating status. (**a**) ExVial after refeeding for 4 h measured with all six dyes (1% w/v) in 2Y10S3C media increased significantly in GL females after 18 h of starvation and was affected by the dye used (two-way ANOVA; starvation, p < 0.0001; dye, p < 0.0001; interaction, p = 0.0058; n = 8). ExVial measured with all six dyes was greater in starved compared to fully fed flies (Bonferroni’s, p = 0.0405 to < 0.0001). (**b**) Mating status and the dye (1% w/v) used affected 24 h ExVial in GL females provided with 2Y10S3C media (two-way ANOVA; mating, p < 0.0001; dye, p < 0.0001; interaction, p < 0.0001; n = 8). Mated females had greater ExVial than virgin females when measured with Orange 4, Yellow 6 and Light Green SF (*Bonferroni’s, p = 0.0017 to < 0.0001), but not with Patent Blue (p = 0.3412) or Acid Blue 3 (p = 0.4745).

A period of starvation increases subsequent consumption of food medium in flies^14^ and mated females consume more food medium than do virgin females^14,16^. ExVial determined with all six lead candidate dyes was increased in mated control females after they were starved (Fig. 2a), indicating that all six dyes detect the effect of prior starvation. ExVial values determined with Orange 4, Yellow 6, Yellow 10 and Light Green SF were greater in mated than in virgin females (Fig. 2b), whereas this effect of mating status was not detected by studies using Patent Blue or Acid Blue 3 (Fig. 2b). Orange 4, Yellow 6, Yellow 10 and Light Green SF might therefore have greater utility in, and be more suitable for, Con-Ex studies than are Patent Blue and Acid Blue 3.

**Figure 3:**
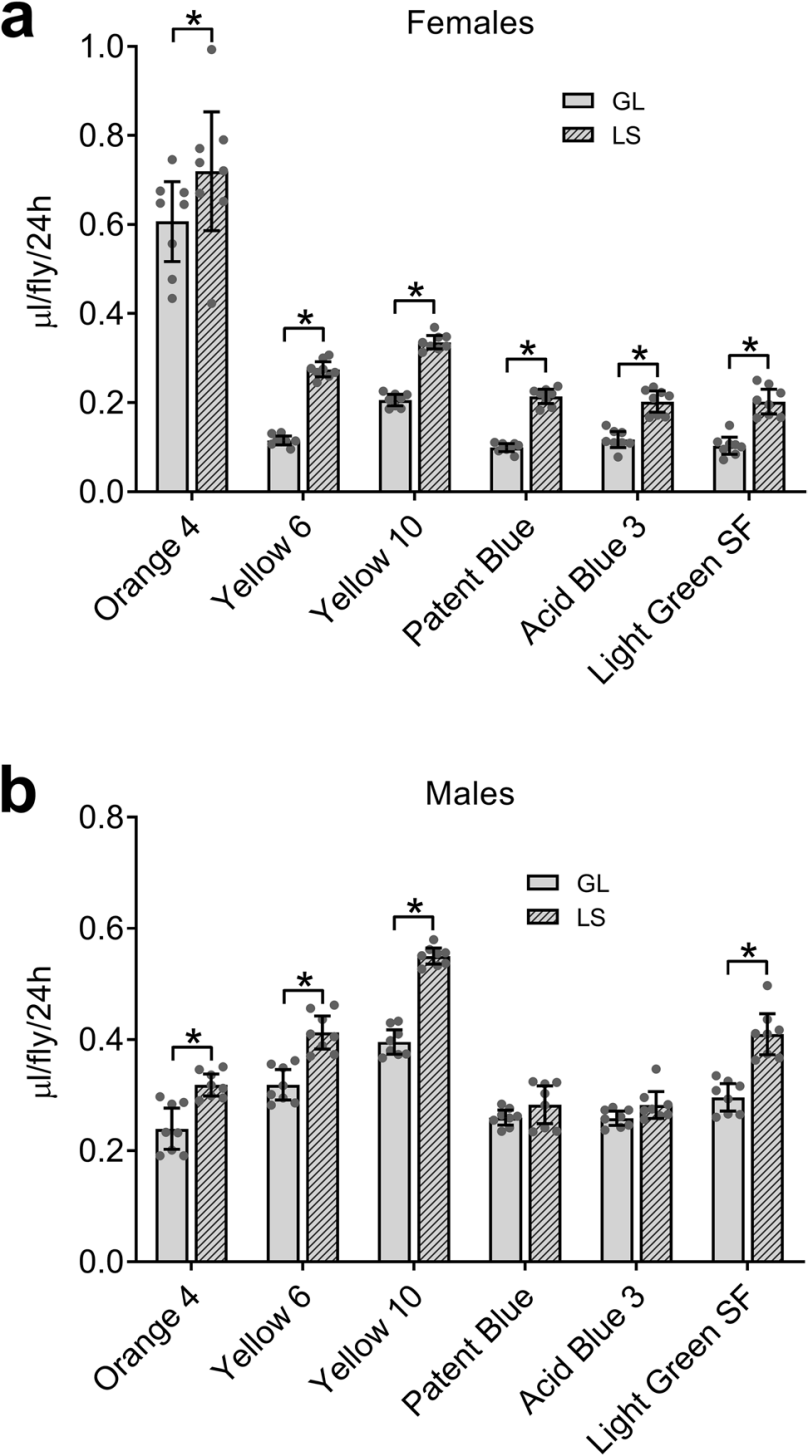
Six lead dyes in Con-Ex: detection of strain effects in females and males. (**a**) Overall, ExVial (24 h feeding on 2Y10S3C media) was greater in LS than GL females and the dye (1% w/v) used had a significant effect (two-way ANOVA; strain, p < 0.0001; dye, p < 0.0001; interaction, p = 0.5708; n = 8). LS females had greater ExVial than GL females with all six dyes (*Bonferroni’s, p = 0.0288 to < 0.0001). (**b**) Males. Strain and the dye (1% w/v) used had significant overall effects on 24 h ExVial in male flies feeding on 2Y10S3C media (two-way ANOVA; strain, p < 0.0001; dye, p < 0.0001; interaction, p < 0.0001; n = 8). ExVial was greater in LS compared to GL males when determined with Orange 4, Yellow 6, Yellow 10 and Light Green SF (*Bonferroni’s, p < 0.0001), but not with Patent Blue (p = 0.8384) or Acid Blue 3 (p = 0.7891).

Lausanne-S (LS) flies have greater ExVial than do GL flies in Con-Ex studies with Blue 1 as a tracer^14^. To determine if the six lead candidate dyes could also detect this strain difference, we assessed ExVial in GL and LS females (Fig. 3a) and males (Fig. 3b). In females, ExVial determined with all six lead candidate dyes was greater in LS than in GL flies as previously reported in studies using Blue 1^14^. In males, ExVial measured with Orange 4, Yellow 6, Yellow 10 and Light Green SF was greater in LS than in GL flies, but this effect of strain was not detected with Patent Blue and Acid Blue 3 (Fig. 3b). We therefore continued characterizing Orange 4, Yellow 6, Yellow 10 and Light Green SF as lead candidate dyes, and did not consider Patent Blue and Acid Blue 3 further.

**Figure 4:**
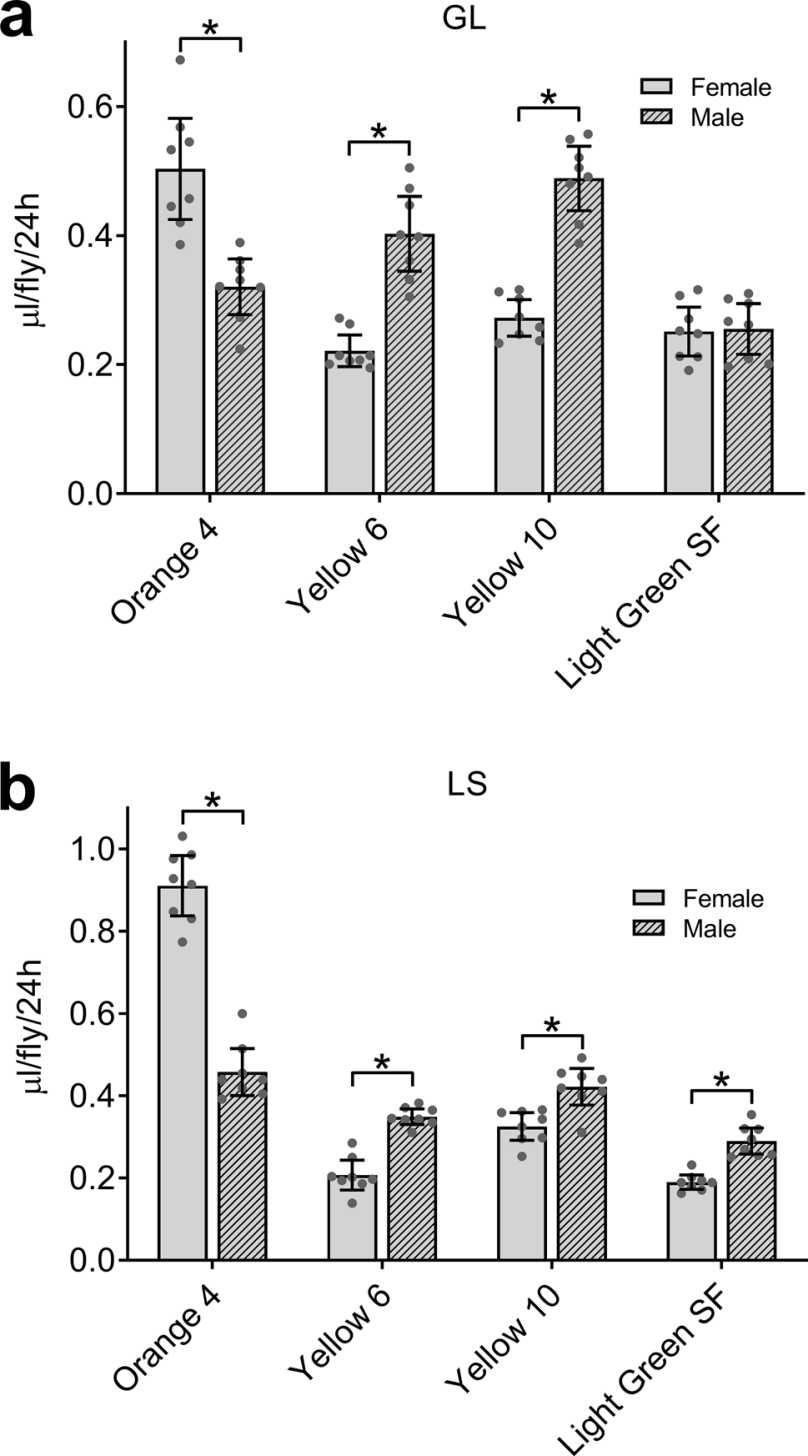
Four lead dyes in Con-Ex: effect of sex in two control strains. ExVial in GL (**a**) and LS (**b**) males and females measured after 24 h with the indicated dyes (1% w/v) in 2Y10S3C media. Sex and the dye used significantly affected ExVial (independent two-way ANOVAs; A: sex, p = 0.0003; dye, p < 0.0001; interaction, p < 0.0001; B: sex, p = 0.0300; dye, p < 0.0001; interaction, p < 0.0001; n = 8). ExVial was significantly affected by sex in both strains as measured by all four dyes (*Bonferroni’s, p = 0.0016 to < 0.0001) except for Light Green SF in GL flies (panel A, p > 0.9999).

Previous studies indicate that female flies consume more food medium than do male flies^14,16^. As expected, ExVial determined with Orange 4 was greater in mated females than in males in both the GL (Fig. 4a) and LS (Fig. 4b) strains. For reasons that are unclear, ExVial determined with Yellow 6, Yellow 10 and Light Green SF were either greater in males than in females or were the same in both sexes in the GL (Fig. 4a) and LS (Fig. 4b) strains. In an independent experiment, we confirmed that ExVial determined with Orange 4 (as in Fig. 4a) and with Blue 1 (as we previously showed^14^) was greater in GL females than males, and that ExVial measured with Yellow 6 and Yellow 10 was greater in males than in females (Supplementary Fig. S3). While we are currently exploring why ExVial measured with Yellow 6 and Yellow 10 is greater in males than in females, we continued characterizing Orange 4 for purposes of this study.

**Figure 5:**
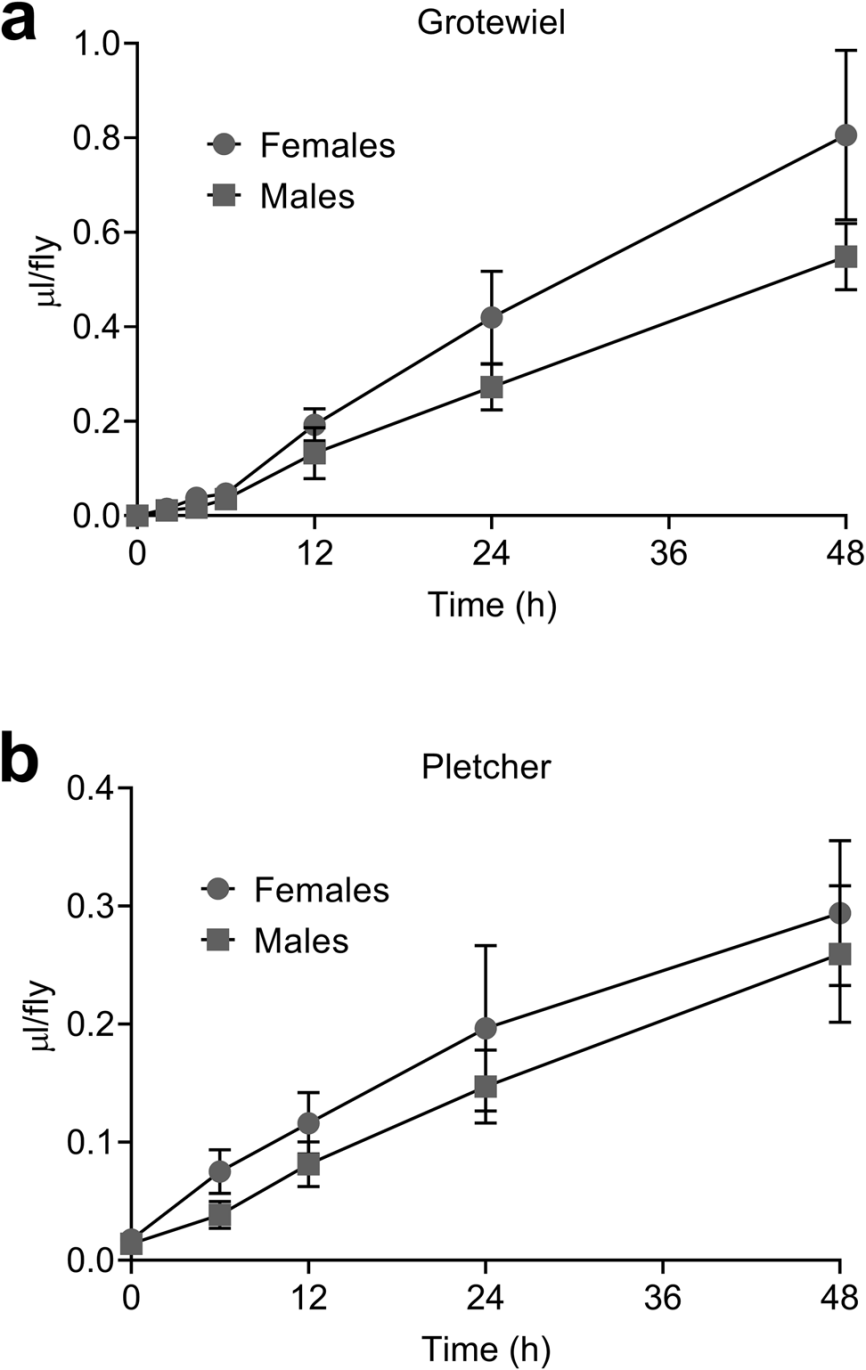
Con-Ex time-courses in control females and males in two different laboratories using two different media. Con-Ex with Orange 4 (1% w/v) in media increased linearly out to 48 h in males and females determined in the Grotewiel (**a**) and Pletcher (**b**) laboratories. (**a**) ExVial from GL females and males consuming 2Y10S3C medium (linear regression: females, R^2^ = 0.8968; males, R^2^ = 0.9431; n = 8 for each time-point). (**b**) ExVial from 10 females and males consuming 10% sugar-yeast medium (linear regression: females, R^2^ = 0.8623; males, R^2^ = 0.7709, n = 8 for each time-point). Slopes derived from linear regression are significantly non-zero in all cases (p < 0.0001). Females consumed more than males in both the Grotewiel (**a**) and Pletcher (**b**) studies (independent two-way ANOVAs; sex, p ≤ 0.0027; time, p < 0.0001).

ExVial values should increase linearly with time when determined with suitable dyes such as Blue 1^14^. Additionally, ExVial determined with dyes such as Blue 1 should be a reliable measure in multiple laboratories^14^. Consistent with these expectations, ExVial increased linearly with time out to 48 h in GL females and males (Fig. 5a, Grotewiel laboratory, Virginia Commonwealth University, 2Y10S3C medium) and in Canton-S females and males (Fig. 5b, Pletcher laboratory, University of Michigan, 10% sugar-yeast medium). Differences in the absolute volumes consumed-excreted in the Grotewiel and Pletcher laboratories are likely due to differences in genetic background and food media used. As we previously found for Blue 1^14^, the progressive time-dependent increase in ExVial measured with Orange 4 as a dye tracer in two different laboratories each using different strains and agar-based media suggests that this dye has utility for Con-Ex studies in a variety of experimental settings.

Flies increase the volume of media consumed in response to decreased concentrations of media components^2,14–16^. Consistent with these previous findings, Con-Ex measured with Orange 4 as a tracer was affected by media dilution, with consumption of 0.25X medium greater than 0.5X or 1.0X media (Supplementary Fig. S4a). Con-Ex with Orange 4 as a tracer was therefore able to detect compensatory feeding like Con-Ex with Blue 1^14,15^.

Statistical power is an important contributor to the practical utility of any experimental method. Using the average mean value for ExVial and the average standard deviation derived from 16 groups using Orange 4 (Figs. 1, 2b, 3, 4, 5, S2-S3) in conjunction with an alpha value of 0.05 (significant p value) and a power of 0.8 (an ability to detect differences in 80% of studies), determining ExVial in Con-Ex studies with Orange 4 as a dye tracer to detect differences of 10%, 20% and 30% between two groups requires 46, 12, and 6 replicates, respectively (Supplementary Fig. S4b). The required number of replicates when using Orange 4 (this report) and Blue 1^14^ as tracers in Con-Ex studies is comparable, although it is higher than in radioactive methods^16^ and that reported for EX-Q^15^. Nevertheless, the number of replicates required to detect 20% or greater differences between two groups using Con-Ex with Blue 1 or Orange 4 as a tracer would be reasonable for most studies.

**Figure 6:**
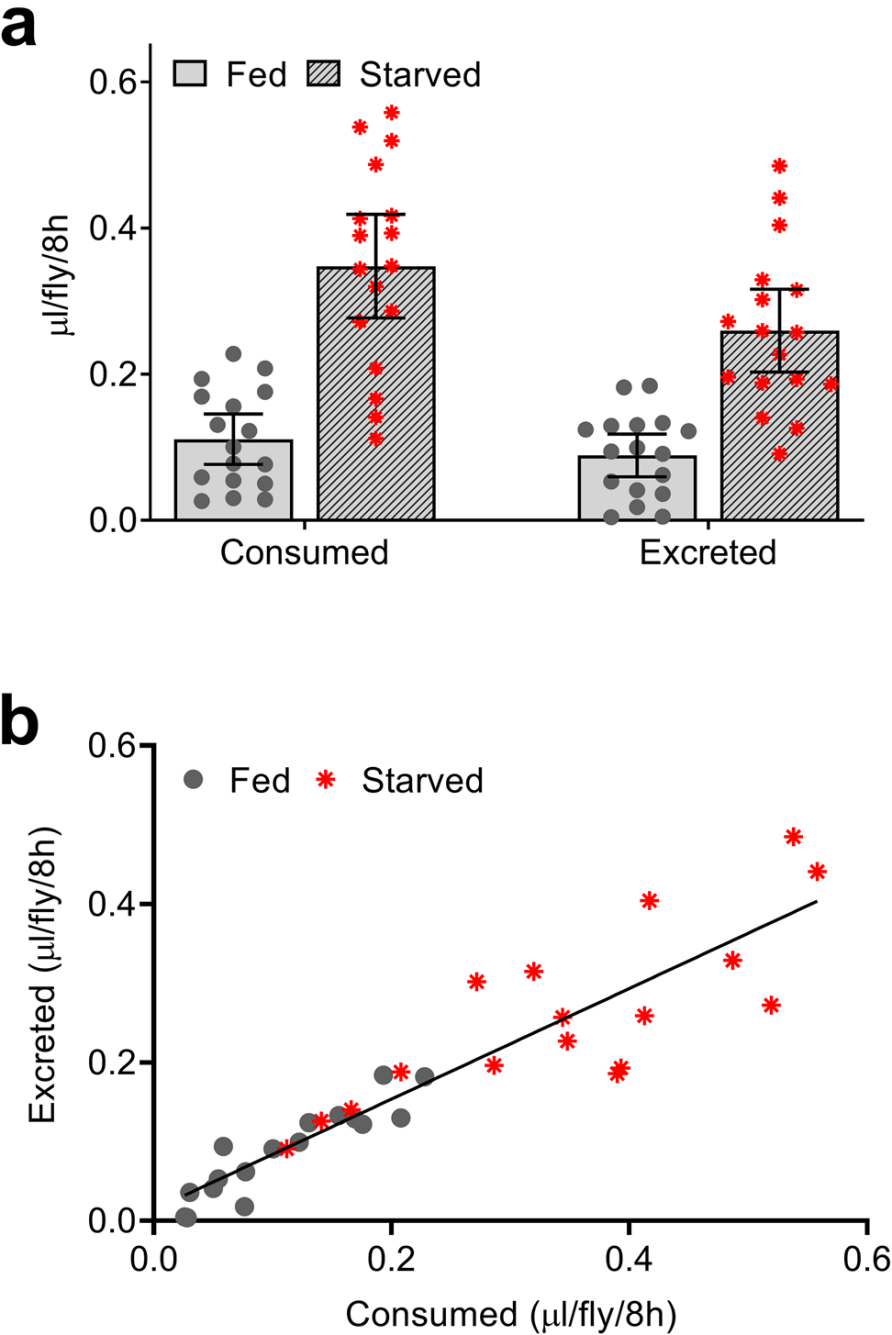
Coupled CAFE:excretion with Orange 4. (**a**) Consumption and excretion of liquid food labeled with Orange 4. GL females were fed standard medium or starved on 1% agar for 18 h, fed 5% sucrose liquid medium labeled with 1% w/v Orange 4 for 8 h via capillary tubes (to measure consumption via CAFE), and then provided with 5% sucrose without dye for 16 to excrete consumed Orange 4 (determined as ExVial + ExPlug). Starvation and measurement method significantly affected the volume of food consumed or excreted (repeated measures two-way ANOVA; matching consumption and excretion values within each vial, p < 0.0001; starvation, p < 0.0001; method, p < 0.0001; interaction, p = 0.0036; n = 17). (**b**) Correlation between consumption and excretion measured in panel A. Each data point represents a single vial. Consumption measurements (X-axis) and excretion measurements (Y-axis) correlated (Pearson r, R^2^ = 0.8395, p < 0.0001, n = 34). Line is best-fit simple linear regression.

We performed coupled CAFE:Con-Ex studies to address whether the volume of Orange 4 excreted by flies reflects the volume of medium consumed. As we reported previously with this approach using Blue 1 as a tracer^14^, (i) the volume of liquid medium consumed was measured by CAFE and the volume of medium excreted was determined as ExVial plus ExPlug (the volume excreted on the plug used to hold CAFE capillary tubes), (ii) flies were either fully fed or starved for 18 h prior to CAFE:Con-Ex experiments to provide a broader range of values for correlation analysis (see below), and (iii) Orange 4 is readily recovered and quantified from the plug (Supplementary Fig. S5). As expected, starved flies had greater CAFE and greater ExVial + ExPlug than fully fed flies (Fig. 6a). The overall volume of Orange 4 consumed via CAFE was greater than the volume of Orange 4 excreted (Fig. 6a), although this difference accounted for only 3.7% of the total variance in these experiments. Importantly, the CAFE and ExVial + ExPlug volumes of Orange 4 strongly correlated (Fig. 6b). Excretion of Orange 4 therefore reflects consumption of the dye, although there does appear to be 20–25% loss of Orange 4 signal after consumption possibly due to metabolism. Nevertheless, the data in Figs. 1, 2, 3, 4, 5, 6 and S1-5 support the use of Orange 4 as a reliable dye tracer for consumption of agar-based food medium in *Drosophila*.

The availability of two dyes suitable for Con-Ex studies (Blue 1^14^ and Orange 4 (this report)) raised the possibility of using Con-Ex to assess food preference in *Drosophila*. In principle, food preference could be determined by concurrently providing flies with 2 different media, each labeled with a different dye tracer, and then measuring the amounts of each dye in the same excretion samples. Peak absorbance of Orange 4 and Blue 1 occurred at distinct wavelengths (Supplementary Fig. S6a), Blue 1 had negligible absorbance at the peak absorbance wavelength of Orange 4 (Supplementary Fig. S6b, S6c) and Orange 4 had essentially no detectable absorbance at the peak absorbance wavelength for Blue 1 (Supplementary Fig. S6d, S6e). Additionally, measured at two dye concentrations, detection of Orange 4 (via absorbance at its peak wavelength) was not compromised by inclusion of Blue 1 (Supplementary Fig. S6b, S6c) or vice versa (Supplementary Fig. S6d, S6e). Orange 4 and Blue 1 can therefore be measured independently and concurrently in samples containing both dyes. Finally, standard curves in water and phosphate-buffered saline (pH 7.6) were indistinguishable as were ExVial absorbance values and ExVial volumes with both Orange 4 and Blue 1 (Supplementary Fig. S7), indicating that either water or neutral-buffered saline can be used in these studies.

While completing our validation studies on Orange 4 (Figs. 1, 2, 3, 4, 5, 6), Yang and co-workers described a modified version of Con-Ex called Excreta-Quantification (EX-Q) for measuring consumption of agar-based food media^15^. The principle operational difference between Con-Ex and EX-Q is the food medium is placed into a feeder cap that covers the open end of the vial in Con-Ex whereas the food medium is in a smaller vessel in EX-Q^15^. This difference allows for more of the excreted dye to be captured and measured in EX-Q compared to Con-Ex^15^.

**Figure 7:**
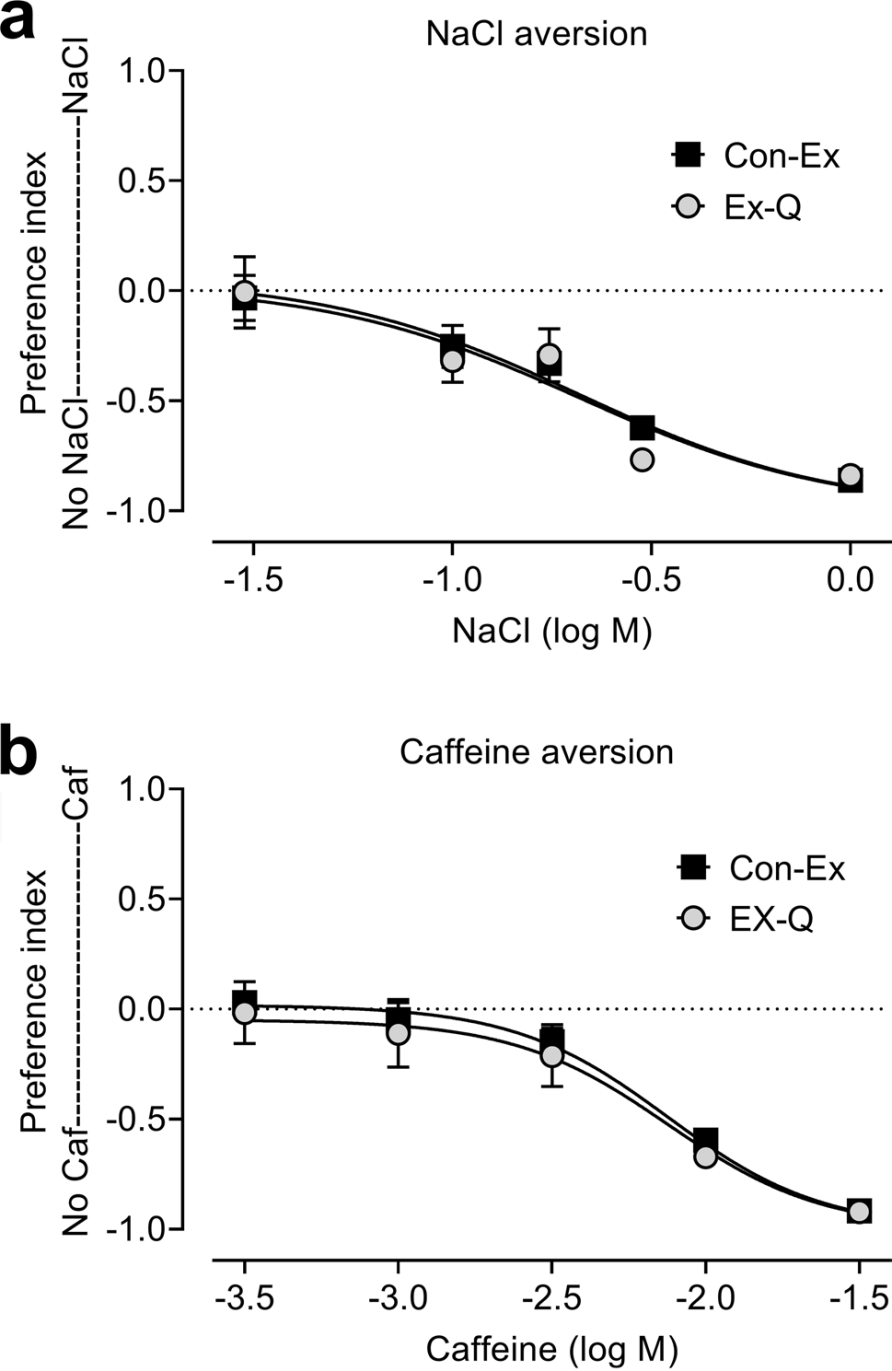
Con-Ex and EX-Q in food preference: preference index. Preference indexes determined from the data in Supplementary Figs. 7 and 8. Con-Ex and EX-Q both detected aversion to NaCl (**a**) and caffeine (**b**) and there were no differences in slopes or IC_50_ values due to the method used (**a**, NaCl, p = 0.4458, common IC50 value = 208 mM; **b**, caffeine, p = 0.6899, common IC50 value = 7.5 mM; n = 16).

We incorporated EX-Q into this project with the goal of performing an initial comparison to Con-Ex in food preference studies. For consistency, we used the same medium (10% sugar, 5% yeast, 10S5Y) as Yang and co-workers in their initial development of EX-Q^15^. In studies using both Con-Ex and EX-Q, we provided flies with concurrent access to 10S5Y food medium labeled with Orange 4 or Blue 1 (each at 1% w/v) in separate vessels within each vial, allowed flies to consume-excrete media from both sources, and then measured the excreted Orange 4 and Blue 1 in the same samples from each vial. For both Con-Ex and EX-Q, flies consumed-excreted Orange 4 and Blue 1 determined as ExVial and did not exhibit a substantial preference (quantified as a preference index, see “Materials and methods”) for either dye (Supplementary Figs. S8a, S9a). Addition of increasing concentrations of the aversive tastants NaCl or caffeine^18,19^ to food labeled with Orange 4 or Blue 1 caused progressive shifts toward excretion of and preference for the media without tastant (Fig. 7a, b, data compiled from individual experiments shown in Supplementary Figs. S8b-S8e and S9b-S9e). The progressive shifts in preference index reflecting NaCl and caffeine aversion measured with Con-Ex and EX-Q were qualitatively similar (Fig. 7a, b). Furthermore, the respective concentrations of NaCl and caffeine required to shift the preference index half-way toward the media without tastant (inhibitory concentration 50, IC_50_) were indistinguishable when determined using Con-Ex and EX-Q (Fig. 7a, b). Our results suggest that both Con-Ex and EX-Q, with Blue 1 and Orange 4 as food tracers, have comparable utility for assessing food preference in response to aversive tastants. Finally, as reported by Yang and co-workers^15^, excreted dye measured by EX-Q was greater than determined by Con-Ex (Supplementary Fig. S8), but this difference in the magnitude of the ExVial dye signal did not have a significant effect on NaCl or caffeine aversion (Fig. 7a, b).

## Discussion

The *Drosophila* model has considerable utility for investigating molecular-genetic, neurobiological, dietary and environmental influences on feeding behavior and physiology. Flies are reared and housed on solid agar-based media in most studies, resulting in a need for methods to assess media consumption under ostensibly standard housing conditions. We previously showed that Blue 1 works well as a dye tracer to track consumption of agar-based media in *Drosophila* in Con-Ex studies^14^. We reasoned that identifying an additional dye tracer would expand the utility of Con-Ex by raising the possibility of (i) using an additional dye to confirm effects on feeding behavior determined with a single dye like Blue 1 and (ii) developing a dye that when paired with Blue 1 would be suitable for food preference studies. The studies reported here identify Orange 4 as a dye suitable for use in Con-Ex experiments and show that this dye has comparable utility to Blue 1.

Interestingly, of the 38 dyes we screened for use in Con-Ex^14^ and this report), only Blue 1 and Orange 4 detect the anticipated effects of starvation, mating status, strain and sex and have other key features such as a lack of effect on consumption at workable concentrations as well as linear accumulation in excretion products over time. In the absence of formal validation of dyes in feeding assays, there is a risk of the tracer not being suitable for detecting meaningful differences between groups, the method lacking sufficient statistical power, unanticipated interactions between the dye and various food media, or the possibility that the dye itself could affect media consumption. Validation of dyes in Con-Ex and similar feeding methods, although time-consuming, is therefore critical for establishing methods suitable for measuring solid food intake in flies.

In the studies we report here, the amount of Orange 4 we recovered in excretion products was 20–25% less than the amount of liquid Orange 4 consumed (measured via CAFE). Whether this loss of Orange 4 is due to breakdown of the dye in the fly digestive tract, incorporation of the dye into internal fly tissues, or some other phenomenon is currently unknown. Importantly, the 20–25% reduction in Orange 4 excreted is not a major limitation to its utility as a dye tracer when viewed within the context of limitations associated with other tracers and methods. Although we did not observe a difference between consumed liquid and excreted Blue 1 previously^14^, flies unavoidably excrete dye on the food medium in Con-Ex studies^14^ and the fraction of the dye excreted on the medium is extremely challenging to directly quantify. Additionally, in studies using internal accumulation of [α-32P]dCTP, approximately 10% of the radioactive tracer is excreted and therefore not typically measured^16^. Although none of the tracers or tracer methods described to date formally quantify total consumption of agar-based media, methods using validated tracers like [α-32P]dCTP, Blue 1 and Orange 4 have excellent utility for estimating consumption and therefore assessing the effects of genetic and environmental manipulations on feeding behavior.

One of the major motivations for identifying and validating an additional dye for Con-Ex was to explore the possibility of pairing it with Blue 1 to assess food preference. The collection of genetic tools and the detailed understanding of the central and peripheral nervous systems in flies make them extremely well suited for investigating the genetic and neurobiological mechanisms underlying food preference or choice, a phenomenon with major implications for human health and disease^17^. Blue 1 and Orange 4 can be measured independently in the same extracts. This makes it possible to provide flies with two kinds of media simultaneously, one labeled with Blue 1 and the other labeled with Orange 4, and then independently detect both dyes in the excretion products. As reported previously in studies with solid media^18,19^, we found that caffeine and NaCl were aversive tastants in food preference studies using Blue 1 and Orange 4 as a dye pair in Con-Ex experiments. We also found that caffeine and NaCl were aversive tastants in EX-Q studies with Blue 1 and Orange 4 as a dye pair, and that aversion to these two tastants was indistinguishable when measured with Con-Ex or EX-Q. Our studies indicate that Orange 4 and Blue 1 work well as a dye pair in food preference studies with aversive tastants in both Con-Ex and EX-Q, that Con-Ex and EX-Q provide comparable results in food preference experiments with aversive tastants, and raise the possibility that this dye pair might also work well in food preference studies with appetitive tastants or other media variations.

Future studies using Blue 1 and Orange 4 as a dye pair in food preference studies could address a large number of important hypotheses and experimental questions. For example, in studies in which flies are allowed to choose between a carbohydrate and a protein source (which alters life span)^22^, it would be informative to know the relative consumption of the two food sources and how that relative consumption affects life span. Additionally, providing flies with a high sugar diet leads to increased consumption^10^, making it important to understand whether consumption of a high sugar diet might alter subsequent food preference. It would also be informative to assess whether juvenile dietary manipulations (which affect mating, fecundity and olfactory responses in adults^23^, impact food preference in adults. Furthermore, given that activation of allatostatin-A^24^ or Taotie neurons alters food consumption in flies^25^, it might be possible to use the *Drosophila* model to comprehensively investigate the neurobiological underpinnings of food preference using the methods described here. Understanding the environmental, neurobiological and molecular-genetic mechanisms that govern food preference would help address the fundamental relationships between food choice, the quantity of consumption, the underlying mechanisms, and human disease states associated with diet.

## Materials and methods

### Test sites

Experiments were performed at Virginia Commonwealth University (Grotewiel laboratory) except those in Fig. 5b which were performed at the University of Michigan (Pletcher laboratory). The materials and methods described correspond to those in the Grotewiel laboratory except for those noted for the University of Michigan.

### Fly husbandry and fly stocks

Flies (*Drosophila melanogaster*) were reared to adulthood on a standard fly Grotewiel laboratory medium (2% yeast, 10% sucrose, 3% cornmeal, and 1% agar with chloramphenicol, ampicillin and tetracycline, 2Y10S3C) at 25 °C/60% relative humidity under a 12:12 h light:dark cycle (lights on, 7:00 a.m.) as previously described^14^. GL, the standard control strain in the Grotewiel laboratory, was generated by backcrossing the w^+^ allele from Canton-S into a *w*^*1118*^ isogenic background strain contributed by Michael Ashburner (BDSC; stock #5905). The GL stock is also reported as r[A]^14^ or *w*^+^^26^. Lausanne-S (LS) flies were from the BDSC (stock #4268).

### Absorbance spectra

Absorbance spectra of samples of Orange 4 or Blue 1 diluted in water at the concentrations indicated in Figure S6 were obtained using a Pharmacia Biotech spectrophotometer (Ultraspec 2000) at 10 nm wavelength increments in polystyrene cuvettes with a 1 cm light path. Absorbance of individual dyes or dye mixtures were obtained similarly using the dye concentrations indicated in Figure S6 at wavelengths producing maximum absorbance.

### Consumption and excretion of dye-labeled media

All comparisons were made between flies reared, housed and tested side-by-side to account for day to day variability in behavior. Flies were grown to adulthood on 2Y10S3C medium, collected under brief (i.e. < 5 min) CO_2_ anesthesia, sorted by sex or mating status, and placed immediately in vials with dye-labeled media to initiate experiments. All studies were performed at 25 °C/60% relative humidity under a 12:12 h light:dark cycle. All experiments were initiated between 10:00 and 11:00 a.m. In all cases, a vial of flies corresponded to a single datum (i.e. n = 1).

Dyes, obtained from the suppliers in Table S1 (provided as supplementary information), were dissolved in media at the concentrations (w/v) indicated in the main text. For Con-Ex studies, agar-based media were poured into feeder caps (FCS13/16NA1; MOCAP, Park Hills, MO) or feeder caps of the same dimensions containing a divider to produce two reservoirs for different media (custom 3-D printed in the Pletcher laboratory using a Formlabs Form 3 printer; www.formlabs.com). For EX-Q studies, agar-based media were poured into inverted 1 ml pipet tips. Media for Con-Ex and EX-Q studies were allowed to cool overnight at 4 °C and warmed to room temperature before the start of each experiment.

Con-Ex studies were performed as described^14^. Briefly, 15 three-five day-old adult flies were placed in each empty food vial, a feeder cap containing dye-labeled media was placed in the top of each vial, flies were allowed to consume-excrete media for the indicated amount of time (typically 24 h), the feeder cap and the flies were discarded, the excretion products were collected in 3 ml of distilled water, absorbance values of the collected excretion products were obtained using a spectrophotometer (Pharmacia Biotech Ultraspec 2000) at the wavelengths indicated in the Figures and Table S1, and the volume of dye excreted in the vial (ExVial) was calculated by interpolation from a standard curve of each dye. The absorbance of fly excreta in the absence of a dye label was negligible at the wavelengths used to measure Orange 4 (0.0028 ± 0.0011), Yellow 6 (0.0029 ± 0.0011), Yellow 10 (0.0034 ± 0.0012), Lightgreen SF (0.0019 ± 0.0010), Patent Blue (0.0019 ± 0.0010) and Acid Blue 3 (0.0019 ± 0.0011) and was therefore disregarded (data are mean ± standard deviation, n = 8).

When used, starvation was achieved by housing flies in vials with 1% agar as a water source and no other food components for 18 h. Flies were refed dye-labeled media for 4 h in Con-Ex experiments to capture the effects of starvation as described or for 8 h in coupled CAFE-excretion studies to produce vial to vial variance necessary for correlation analyses^14^.

Coupled CAFE-excretion studies were performed as described using capillary tubes (borosilicate glass micropipets, 5 µL, VWR, # 53432-706)^14^. Briefly, flies (10 flies/vial, starved or fully fed) were fed 5% sucrose liquid medium labeled with Orange 4 for 8 h via capillary tubes (to determine CAFE), and then provided with 5% sucrose without dye for 16 h to excrete consumed Orange 4 (determined as ExVial). Recover of Orange 4 from foam plugs used to hold capillary tubes was assessed as described for Blue 1^14^.

Con-Ex food preference studies were performed as described above for Con-Ex except that flies were allowed to consume two different media (one labeled with Blue 1 and the other labeled with Orange 4) from a divided feeder cap, and the amounts of Orange 4 and Blue 1 were determined in the same ExVial samples. NaCl and caffeine (Sigma), aversive tastants^18,19^, were added in increasing concentrations to either the media labeled with Orange 4 or Blue 1 in a counter-balanced design. Preference indexes were calculated for each vial as [volume of media with tastant – volume of media without tastant]/[volume of media with tastant + volume of media without tastant]. In principle, this preference index can vary from + 1 (indicating consumption of only media with tastant) to -1 (indicating consumption of only media without tastant).

We adapted EX-Q methods from Yang and co-workers^15^ for food preference studies. Each empty feeder cap with two 9.4 mm holes bored through the top were retro-fitted with two 1 ml pipet tips containing food media. The larger ends of the tips were positioned toward the inside of the feeder caps (i.e. the fly side) with the rims of the pipet tips approximately 1 mm from the inside surfaces of the feeder cap. Flies (15/vial) were added to an empty food vial, an EX-Q cap fitted with two pipet tips containing two different media (one labeled with Blue 1 and one labeled with Orange 4) was placed in the top of the vial, and the amount of Blue 1 and Orange 4 consumed-excreted was determined after 24 h of consumption as in Con-Ex studies except that dyes on the feeder cap as well as dyes on the inside of the vial was collected as previously described^14^.

### Statistical analyses

Of the 199 groups of results analyzed for this study, 90% (179 groups) did not have significantly non-Gaussian distributions and 10% (20 groups) had non-Gaussian distributions. Given that the vast majority of results did not have non-Gaussian distributions, we used parametric statistical analyses throughout this study^27^. One- and two-way ANOVAs followed by Bonferroni’s multiple comparison tests (hereafter simply Bonferroni’s), non-linear regression (log[inhibitor] vs. response-variable slope, bottom constrained to − 1, followed by Extra sum of squares F-test to compare slopes and IC_50_ values), Pearson correlation, and one-sample t tests (to compare observed values to a theoretical value) were performed using Prism 9.0.1 (GraphPad, San Diego, CA). Whenever possible and unless otherwise noted, results are presented as individual replicates with the mean (as a bar) and error bars representing 95% confidence intervals of the means. Power calculations were performed using a tool available at https://www.stat.ubc.ca/~rollin/stats/ssize/n2.html.

### University of Michigan methods

Canton-S flies were used for Fig. 5B. Flies were maintained on a standard cornmeal-based growth medium at 25 °C with 60% humidity under a 12:12 light dark cycle. Larval density was controlled by aliquoting 32 μl of collected eggs into rearing bottles (www.flystuff.com #8003) containing 25 ml of standard cornmeal-based growth medium. Following eclosion, adult flies were transferred into rearing bottles containing SY10% medium (10% sucrose, 10% yeast) and allowed to mate for two days, after which the sexes were separated under light CO_2_ anesthesia. Ten male or female flies were placed into individual vials (www.flystuff.com #8002) and allowed to age for 5–7 days before Con-Ex studies (performed as described above).

## Supplementary Information

Supplementary Information 1.

Supplementary Information 2.

## Data Availability

The datasets generated during and/or analyzed during the current study are available from the corresponding author on reasonable request.
