# Supplementary material for: Expansion and application of dye tracers for measuring solid food intake and food preference in Drosophila
Source: Sci Rep. 2021 Oct 8;11:20044. doi: 10.1038/s41598-021-99483-7 (PMC8501022; doi:10.1038/s41598-021-99483-7)

**Supplementary information**

**Supplementary Figure 1. Screen of dyes for use in Con-Ex studies.** Data are ExVial absorbance values after 24 hour Con-Ex experiments using mated GL females with each of the indicated dyes at 0.5, 1.0 and 2.0% (w/v) in 2Y10S3C food medium. Red arrows indicate dyes chosen for further study (**a**, Orange 4 and Yellow 10; **b**, Patent Blue and Yellow 6; **c**, Acid Blue 3; **d**, Light Green SF). Data are represented as mean (symbols) ± S.E.M (n=6-8 for each dye at each concentration). Absorbance wavelengths are show in parentheses after each dye.

**Supplementary Figure 2. Con-Ex repeated with expanded Orange 4 concentrations**. Con-Ex in mated GL females (24h feeding) was not affected by Orange 4 concentration in 2Y10S3C media (one-way ANOVA, p = 0.0912, n=8).

**Supplementary Figure 3. Confirmation of interaction between sex and dye in Con-Ex studies.** Sex and dye significantly impacted ExVial (24 h feeding) in GL flies when determined using the four indicated dyes (two-way ANOVA; sex, p = 0.0278; dye, p < 0.0001; interaction, p < 0.0001; n = 8). ExVial in females and males was different when measured with all four dyes (*Bonferroni’s, p = 0.0137 to < 0.0001).

**Supplementary Figure 4. Compensatory feeding and power analysis of Con-Ex with Orange 4.** (**a**) Con-Ex with GL females fed 0.25X, 0.5X or 1.0X of 2Y10S3C media labeled with 1% Orange 4 for 24h. Media concentration affected Con-Ex (one-way ANOVA, p = 0.0002, n=8). Con-Ex with 0.25X was significantly different than 0.5X and 1.0X (*Bonferroni’s, p = 0.0089 and 0.0002, respectively). (**b**) Results of power analysis performed as described in Methods using an average mean value for ExVial of 0.455 µl/fly/24 h and an average standard deviation of 0.077 (derived from 16 groups using Orange 4 (Figs. 1, 2b, 3-5, S2-S3) in conjunction with an alpha value of 0.05 (significant p value) and a power of 0.8 (an ability to detect differences 80% of the time). The numbers of replicates required to detect differences in ExVial of 30, 20 and 10% between two groups are indicated.

**Supplementary Figure 5. Recovery of Orange 4 from foam plugs used in CAFE:Con-Ex studies.** Orange 4 added to foam plugs was quantitatively recovered by water extraction (one-sample t tests to compare recovered to added Orange 4 with alpha correction; 0 µl, p > 0.9999; 1 µl, p = 0.1470; 2 µl, p = 0.4755; n = 8).

**Supplementary Figure 6. Absorbance characteristics of Orange 4 and Blue 1.** Dyes were in solution (w/v in water) at 0.00333% (**a, b** and **d**), 0.00133% (**c**) and (0.001%) (**e**). (**a**) Absorbance spectra for Orange 4 and Blue 1. (**b**-**e**) Absorbance of Orange 4 and Blue 1 do not affect absorbance of the other in mixed solutions. Absorbance at 483 nm for Orange 4 (**b and c**) and 630 nm for Blue 1 (**d and e**) in solutions with Orange 4 alone, Blue 1 alone, or both Orange 4 plus Blue 1. There were significant overall effects of the dyes or dye mixes on absorbance (individual one-way ANOVAs, p < 0.0001, n = 3 in **b** and **d**, n = 8 in **c** and **e**). (**b** and **c**) Orange 4 was readily detected at 483 nm, whereas Blue 1 had minimal absorbance at this wavelength (*Bonferroni’s, compared to Orange 4, p < 0.0001) and Blue 1 did not impact the absorbance of Orange 4 in a mixed solution (Bonferroni’s, compared to Orange 4 alone, p = 0.8717-0.9939). (**d** and **e**) Blue 1, but not Orange 4, had strong absorbance at 630 nM (*Bonferroni’s, compared to Blue 1 alone, p < 0.0001). Absorbance at 630 nm was indistinguishable in solutions of Blue 1 alone and Orange 4 plus Blue 1 (p = 0.6274-0.8581).

**Supplementary Figure 7. Extraction of ExVial with water and neutral-buffered saline in Con-Ex with Orange 4 and Blue 1.** (**a**, **b**) Standard curves with Orange 4 (**a**) and Blue 1 (**b**) in water and phosphate-buffered saline (PBS, pH 7.6) (n = 3). (**c**, **d**) ExVial absorbance values from GL females fed standard media with Orange 4 (**c**) or Blue 1 (**d**) are not different when extracted in water or PBS (individual t tests; Orange 4, p = 0.1478, n = 8; Blue 1, p = 0.7597, n = 8). (**e**, **f**) ExVial volumes (interpolated from panels c and d) from GL females fed standard media with Orange 4 (**e**) or Blue 1 (**f**) were not different when extracted in water or PBS (individual t tests; Orange 4, p = 0.3069, n = 8; Blue 1, p = 0.7205, n = 8).

**Supplementary Figure 8. Con-Ex and EX-Q in food preference: volume data.** Con-Ex and EX-Q values measured when GL females were presented a choice of 10Y5S medium labeled with either 1% w/v Blue 1 or Orange 4. (**a**) Con-Ex and EX-Q with 10Y5S media without added tastants. Method of measurement, but not dye, had a significant effect (two-way ANOVA; method, p = 0.0091; dye, p = 0.0777; interaction, p = 0.1578; n = 8). (**b**-**e**) NaCl and caffeine aversion. Con-Ex and EX-Q from food choice studies with 10Y5S media labeled with tastant added to Orange 4 (**b**, **d**) or Blue 1 (**c**, **e**). NaCl (**b**, **c**) and caffeine (**d**, **e**) were added at the indicated concentrations. (**b**) For Con-Ex, NaCl (in Orange 4) and dye had significant effects (two-way ANOVA; NaCl, p < 0.0001; dye, p < 0.0001; interaction, p < 0.0001; n = 8). Con-Ex for Blue 1 and Orange 4 were significantly different when Orange 4 media contained 100 mM or more NaCl (*Bonferroni’s, p = 0.0197 to < 0.0001). For EX-Q, NaCl and dye had significant effects (two-way ANOVA; NaCl, p < 0.0001; dye, p < 0.0001; interaction, p < 0.0001; n = 8). EX-Q values from Blue 1 and Orange 4 were different at most NaCl concentrations tested (*Bonferroni’s, p = 0.0149 to < 0.0001). (**c**) For Con-Ex, NaCl (in Blue 1) and dye had significant overall effects (two-way ANOVA; NaCl, p < 0.0001; dye, p < 0.0001; interaction, p < 0.0001; n = 8). For EX-Q, NaCl and dye had significant effects (two-way ANOVA; NaCl, p = 0.0341; dye, p < 0.0001; interaction, p < 0.0001; n=8). Con-Ex and EX-Q values for Orange 4 and Blue 1 were different at all NaCl concentrations (*Bonferroni’s, p = 0.0003 to < 0.0001). (**d**) Caffeine (in Orange 4) and dye significantly affected Con-Ex and EX-Q (individual two-way ANOVAs; caffeine, p < 0.0001; dye, p < 0.0001; interaction, p < 0.0001; n=8). Con-Ex for Blue 1 and Orange 4 were significantly different when media labeled with Orange 4 contained 10 mM or more caffeine (*Bonferroni’s, p < 0.0001). EX-Q determination of Orange 4 was greater than Blue 1 when the Orange 4 media contained 0.32 and 1 mM caffeine, and was greater in Blue 1 when the Orange 4 media contained 10 mM or more caffeine (*Bonferroni’s, p < 0.0001). (**e**) Caffeine (in Blue 1) and dye significantly affected Con-Ex and EX-Q (individual two-way ANOVAs; caffeine, p = 0.0112 to < 0.0001; dye, p < 0.0001; interaction, p < 0.0001; n = 8). Con-Ex measurement of Orange 4 and Blue 1 were different when Blue 1 contained 3.2 mM or more caffeine (*Bonferroni’s, p < 0.0001). EX-Q determination of Orange 4 and Blue 1 was different at all concentrations of caffeine (*Bonferroni’s, p < 0.0001).

**Supplementary Figure 9. Con-Ex and EX-Q in food preference: half preference indexes.** Preference indexes determined from the volume data in Supplementary Fig. S8. (**a**) In the absence of added tastants using GL females and 10S5Y media, the preference index determined using Con-Ex and EX-Q were not significantly different (t test, p = 0.2111) and these preference indexes were not significantly different than 0 (one-sample t tests to compare to 0; ExVial, p = 0.4499; EX-Q, p = 0.0614; n = 8). (**b**-**e**) Con-Ex and EX-Q both detected aversion to NaCl (**b**, when in Orange 4; **c**, when in Blue 1) and caffeine (**d**, when in Orange 4; **e**, when in Blue 1). The best-fit non-linear regression model for slope and IC_50_ values for NaCl determined by Con-Ex and EX-Q was not different in panel **a** (F test, p = 0.5943, common calculated IC_50_ = 169 mM), but was different in panel **b** (p < 0.0145; Con-Ex IC_50_, 206 mM; EX-Q IC_50_, 289 mM). The best fit non-linear regression model was not significantly different for Con-Ex and EX-Q in panel **d** (p = 0.1729, common calculated IC50 = 7.4 mM) or in panel **e** (p = 0.2388; common calculated IC_50_ = 7.6 mM).


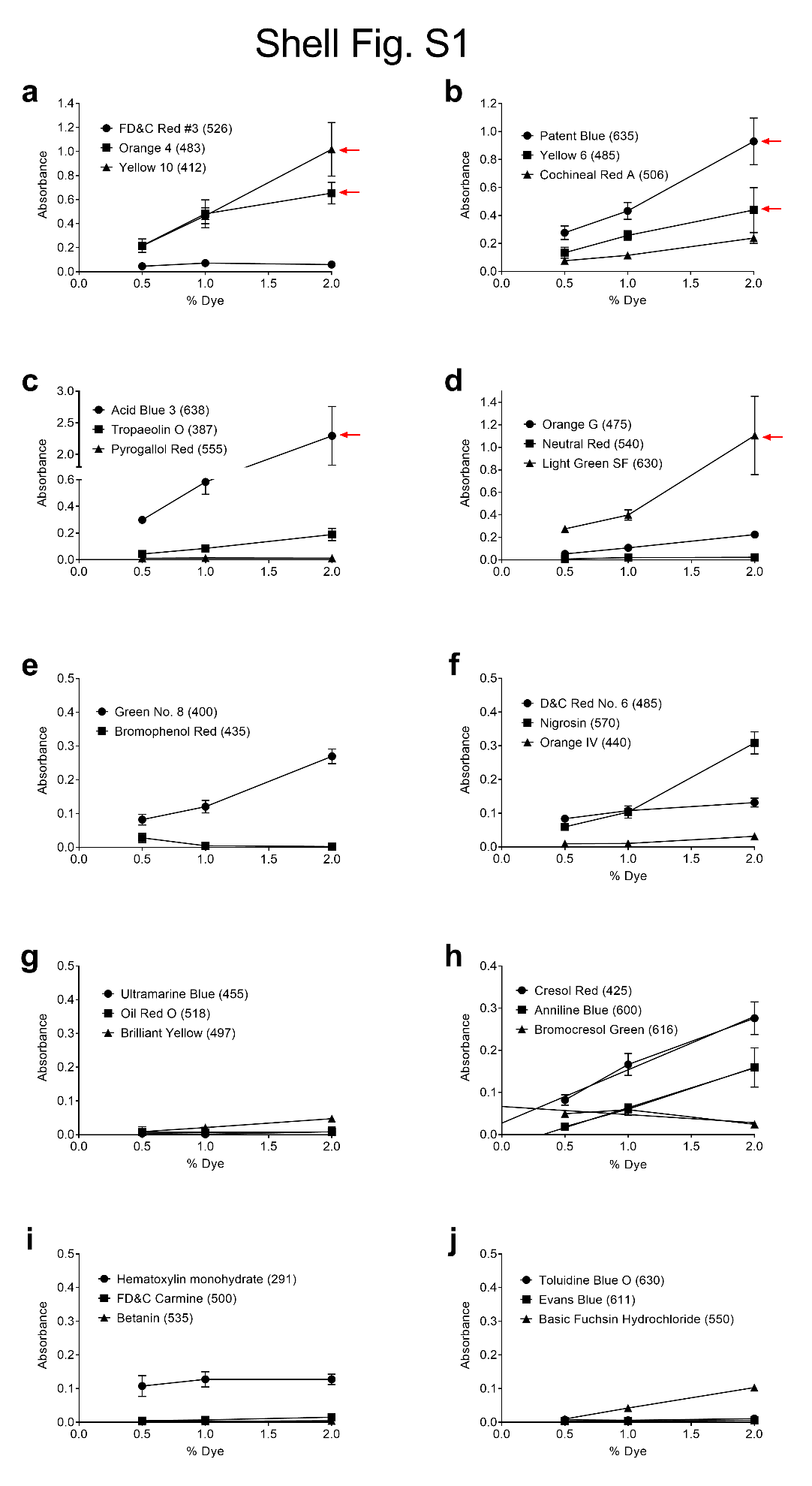


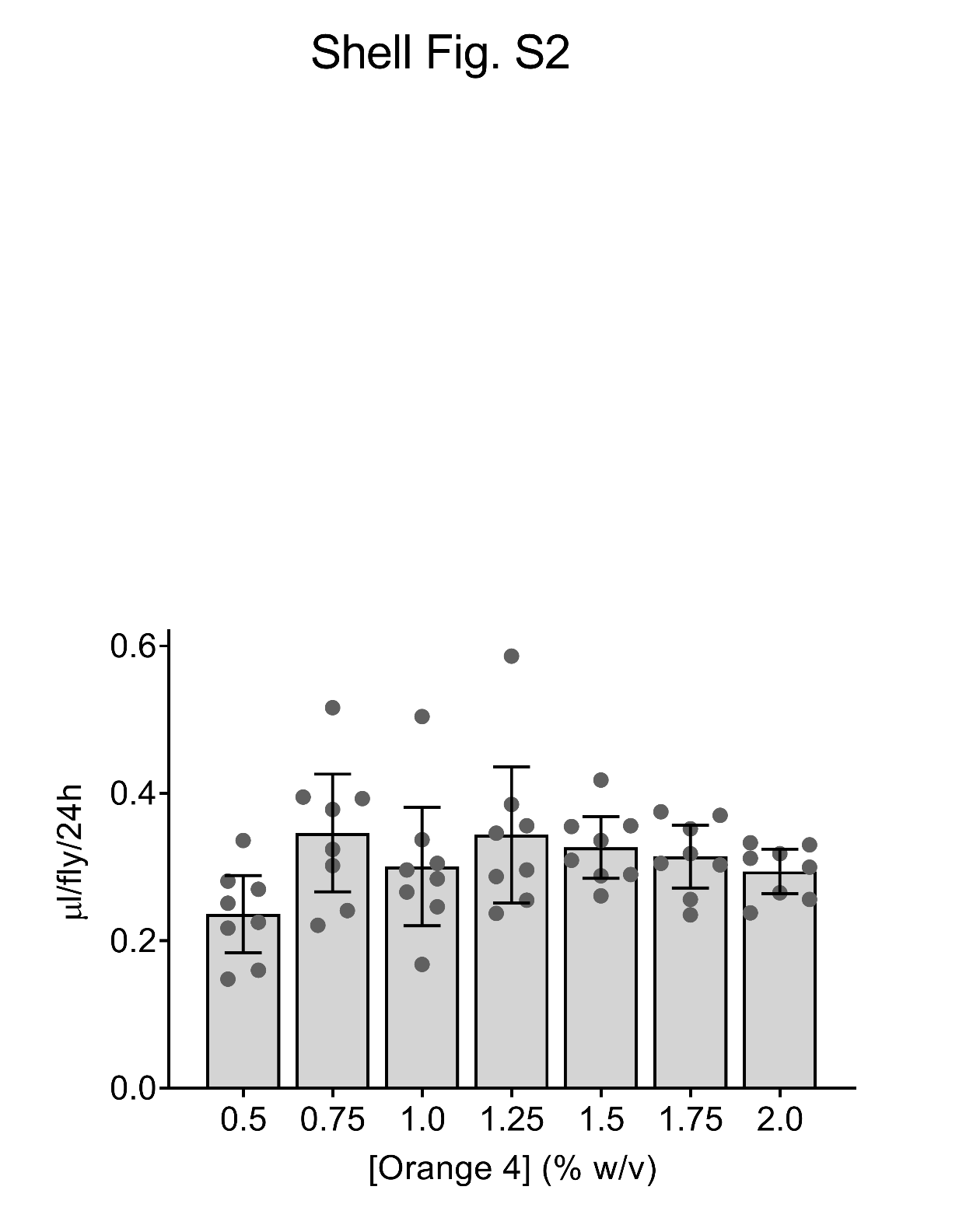


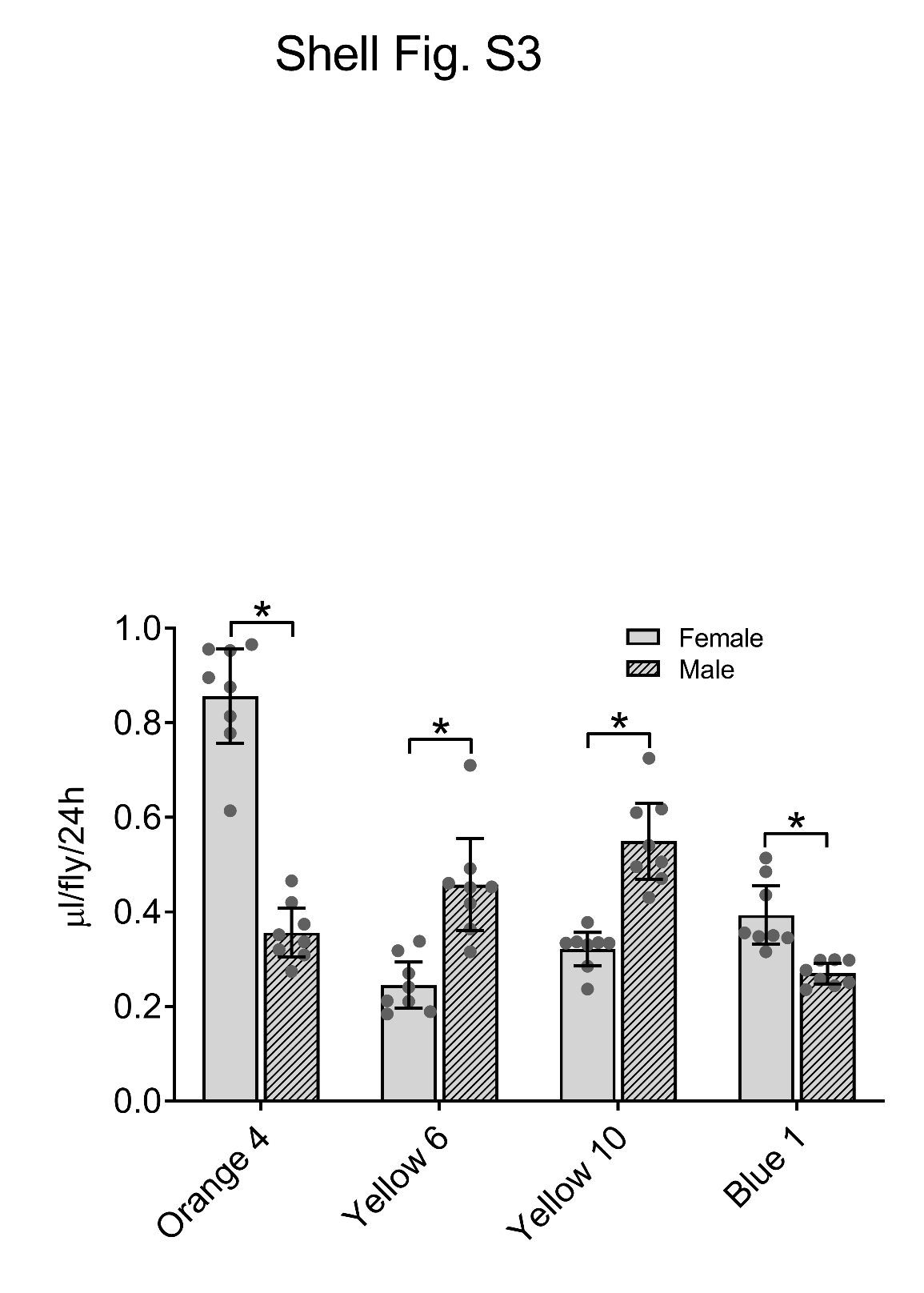


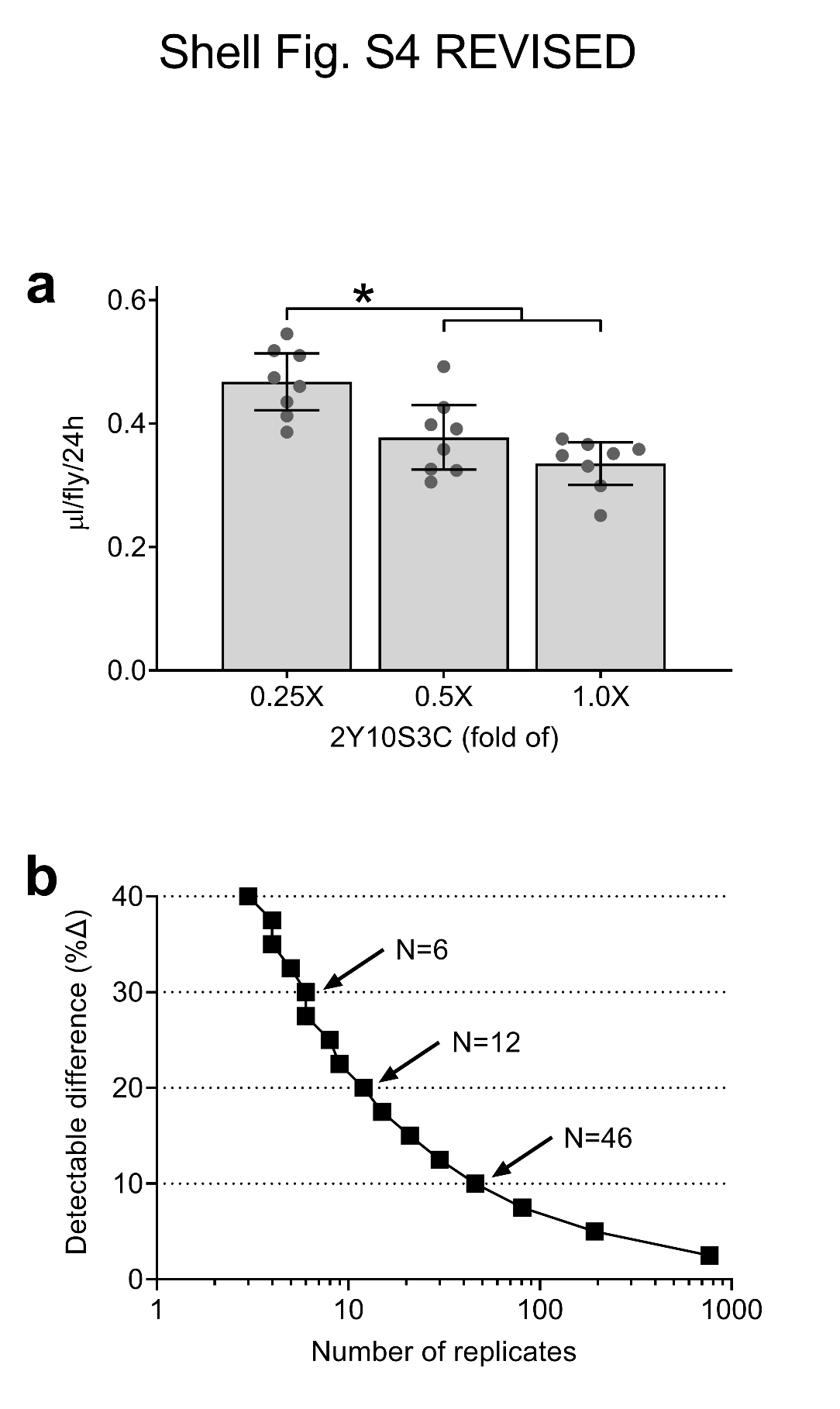


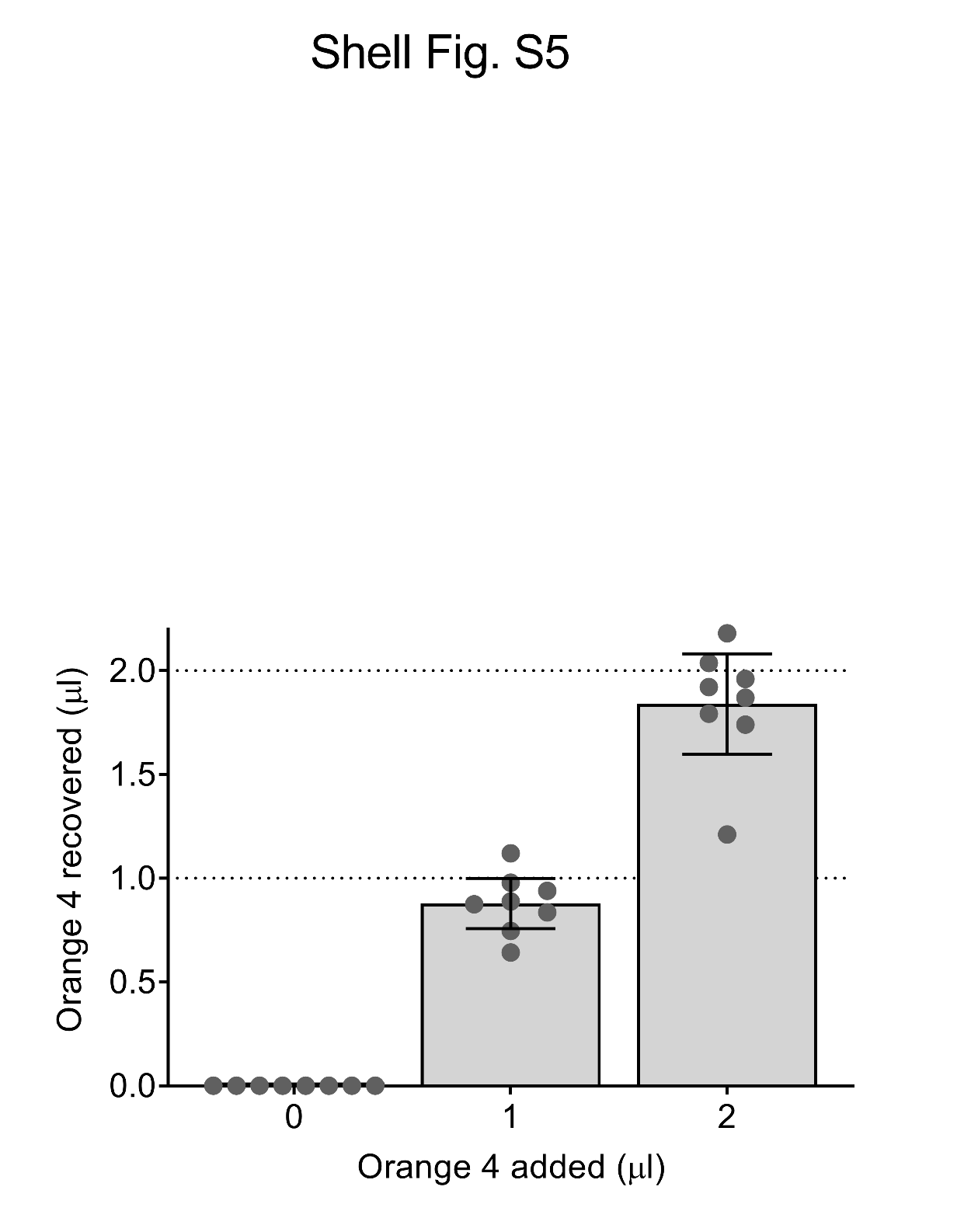


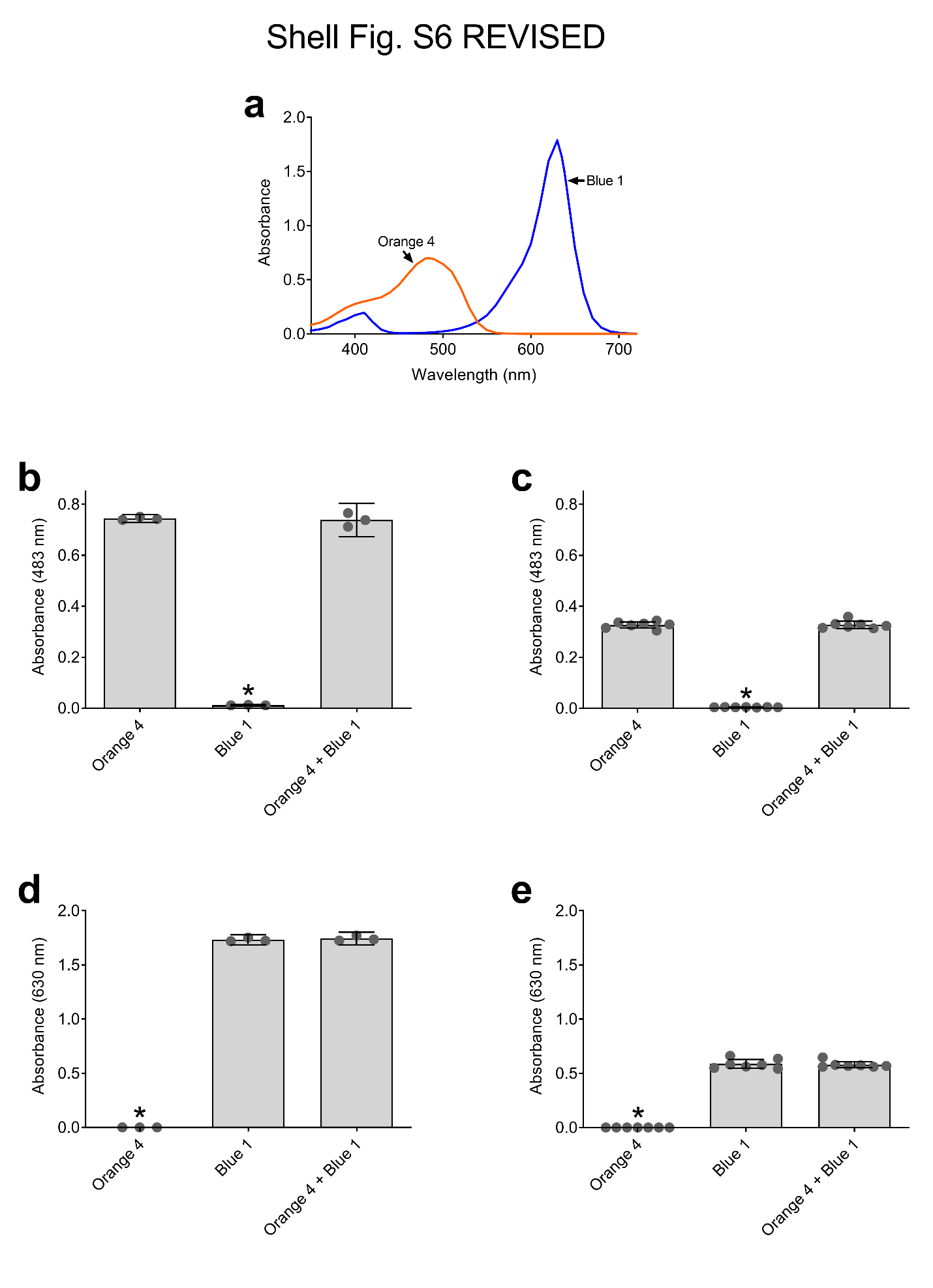


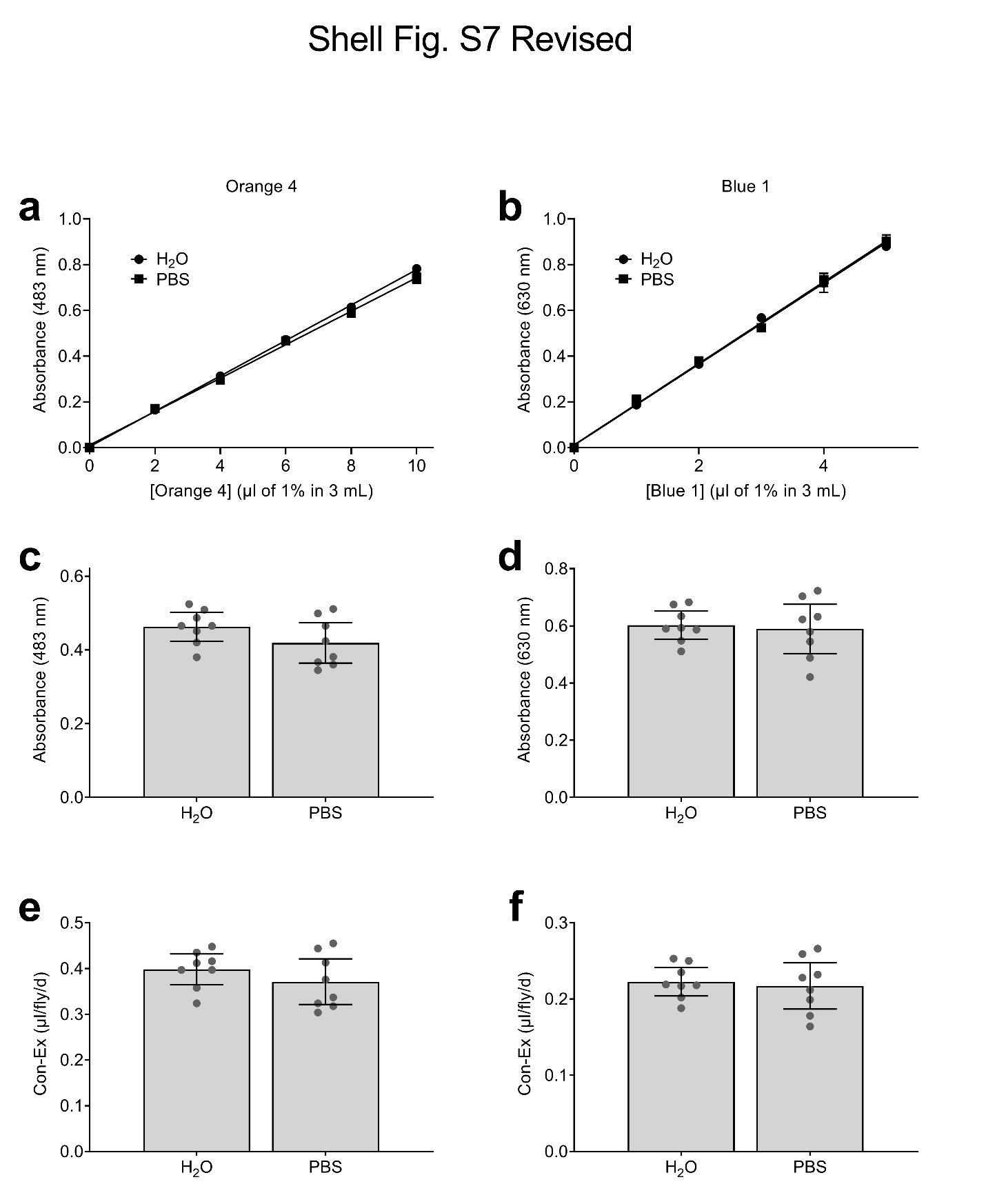


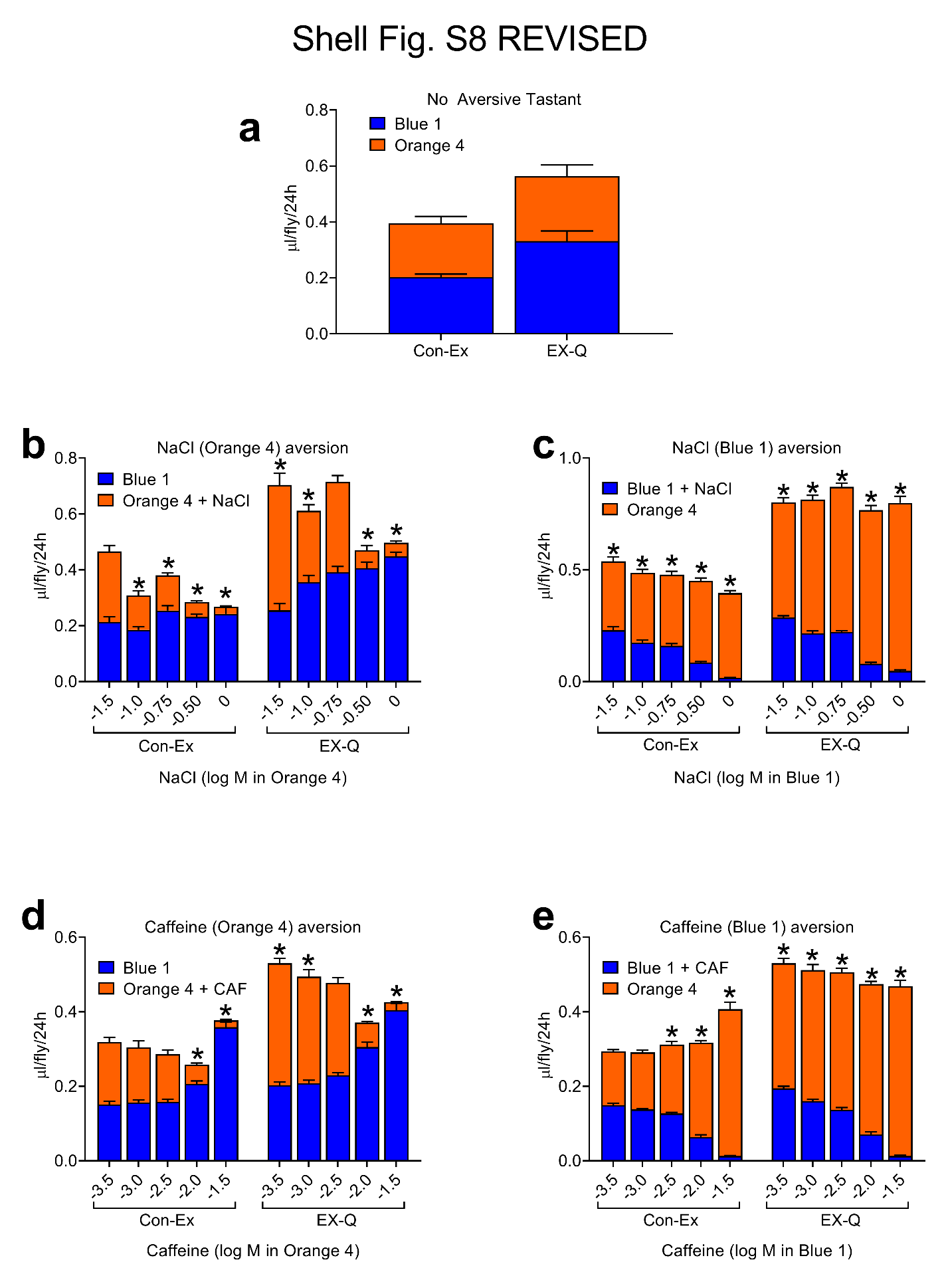


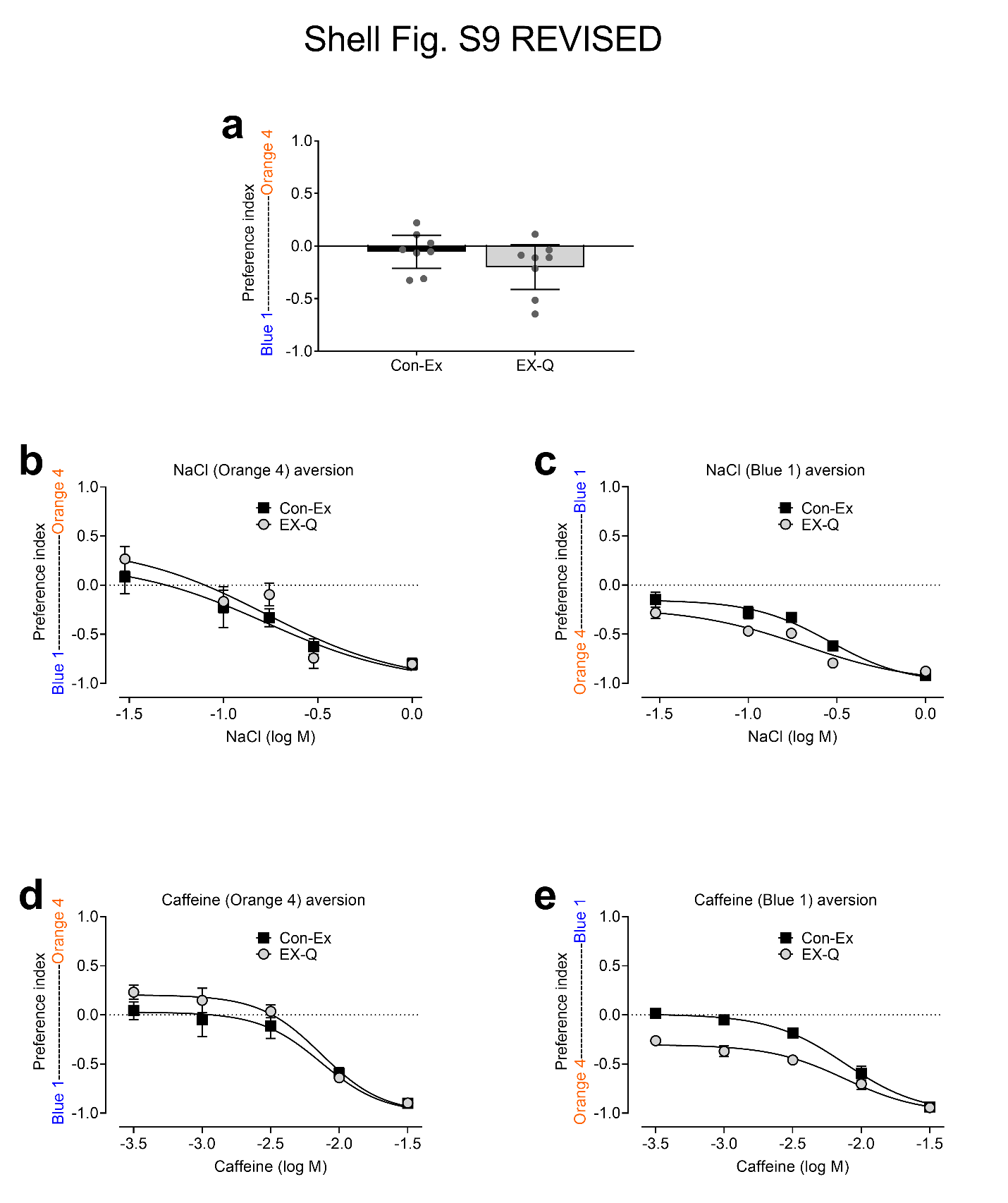

Supplement: Supplementary file 1 — Supplementary Information 1. [file 41598_2021_99483_MOESM1_ESM.docx]
